# IDH1 mutant glioma is preferentially sensitive to the HDAC inhibitor panobinostat

**DOI:** 10.1007/s11060-021-03829-0

**Published:** 2021-08-23

**Authors:** Thomas K. Sears, Craig M. Horbinski, Kevin D. Woolard

**Affiliations:** 1 Department of Neurological Surgery, Northwestern University, Chicago, IL USA; 2 Department of Pathology, Northwestern University, Chicago, IL USA; 3 Department of Pathology, Immunology, and Microbiology, UC Davis School of Veterinary Medicine, Davis, CA USA

**Keywords:** Isocitrate dehydrogenase (IDH) mutant glioma, Histone deacetylase (HDAC) inhibition, Panobinostat, Valproic acid

## Abstract

**Introduction:**

A large subset of diffusely infiltrative gliomas contains a gain-of-function mutation in isocitrate dehydrogenase 1 or 2 (IDH1/2^mut^) which produces 2-hydroxglutarate, an inhibitor of α-ketoglutarate-dependent DNA demethylases, thereby inducing widespread DNA and histone methylation. Because histone deacetylase (HDAC) enzymes are localized to methylated chromatin via methyl-binding domain proteins, IDH1/2^mut^ gliomas may be more dependent on HDAC activity, and therefore may be more sensitive to HDAC inhibitors.

**Methods:**

Six cultured patient-derived glioma cell lines, IDH1^wt^ (n = 3) and IDH1^mut^ (n = 3), were treated with an FDA-approved HDAC inhibitor, panobinostat. Cellular cytotoxicity and proliferation assays were conducted by flow cytometry. Histone modifications and cell signaling pathways were assessed using immunoblot and/or ELISA.

**Results:**

IDH1^mut^ gliomas exhibited marked upregulation of genes associated with the HDAC activity. Glioma cell cultures bearing IDH1^mut^ were significantly more sensitive to the cytotoxic and antiproliferative effects of panobinostat, compared to IDH1^wt^ glioma cells. Panobinostat caused a greater increase in acetylation of the histone residues H3K14, H3K18, and H3K27 in IDH1^mut^ glioma cells. Another HDAC inhibitor, valproic acid, was also more effective against IDH1^mut^ glioma cells.

**Conclusion:**

These data suggest that IDH1^mut^ gliomas may be preferentially sensitive to HDAC inhibitors. Further, IDH1^mut^ glioma cultures showed enhanced accumulation of acetylated histone residues in response to panobinostat treatment, suggesting a direct epigenetic mechanism for this sensitivity. This provides a rationale for further exploration of HDAC inhibitors against IDH1^mut^ gliomas.

**Supplementary Information:**

The online version contains supplementary material available at 10.1007/s11060-021-03829-0.

## Introduction

Mutations in *isocitrate dehydrogenase 1* and *2* (termed IDH1/2^mut^) are present in approximately 25–30% of all diffusely infiltrative gliomas in adults, and are now part of the diagnostic criteria for oligodendrogliomas [1]. Single point mutations at key arginine amino acid residues cause a dramatic change in enzyme activity, where α-ketoglutarate is converted into D-2-hydroxyglutarate (D2HG) [2]. D2HG, in turn, inhibits certain DNA- and histone-demethylating dioxygenases that require α-ketoglutarate as a co-substrate. This gradually leads to a global increase in DNA and histone methylation in IDH1/2^mut^ gliomas [3–5]. While the exact mechanisms connecting this cellular activity to gliomagenesis are not yet completely understood, IDH1/2^mut^ gliomas are generally much less aggressive than their IDH1/2 wild-type (IDH^wt^) counterparts. In fact, the distinction is so dramatic that the new World Health Organization (WHO) scheme has split adult-type infiltrative gliomas into three discrete subsets: “Astrocytoma, IDH1/2^mut^, WHO grades 2–4,” “Oligodendroglioma, IDH1/2^mut^ and 1p/19q-codeleted, WHO grades 2–3” and “Glioblastoma, IDH1/2^wt^, WHO grade 4” [6].

Since IDH1/2^mut^ occurs early in gliomagenesis, and the histone/DNA hypermethylation state of IDH1/2^mut^ gliomas is largely retained even during disease progression [7–10], there is a possibility that epigenetic modifiers may be particularly effective against IDH1/2^mut^ glioma. Histone deacetylases (HDACs) are a class of epigenetic “erasers” that promote chromatin compaction via removal of acetate residues from acetylated histones [11]. HDACs form complexes with methyl-binding domain (MBD) [12] and/or MeCP2 proteins [13], particularly the NuRD complex, which includes HDAC1/2 and MBD2/3 [14–17]. This interaction directs HDAC enzymes to methylated DNA to facilitate chromatin compaction and epigenetic repression of genes associated with these methylated DNA regions. We therefore sought to determine whether IDH1/2^mut^ gliomas might be preferentially sensitive to HDAC inhibitors (HDACi), which are already being used to treat malignancies elsewhere in the body [18].

## Results

### Genes that promote HDAC function are upregulated in IDH1/2mut glioma

**Fig. 1:**
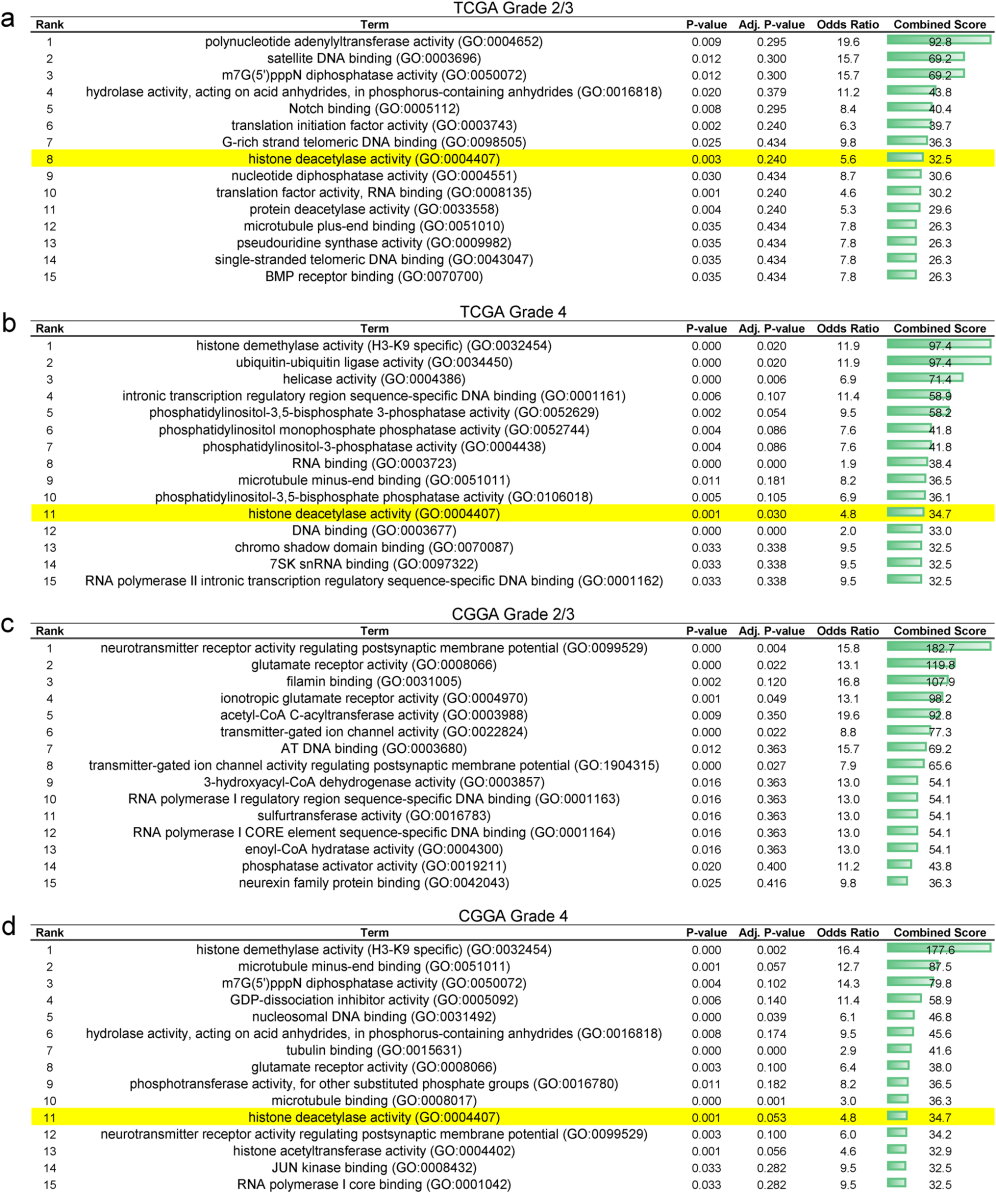
Genes involved in promoting HDAC activity are upregulated in IDH1^mut^ glioma. **a**–**d** Gene ontology (GO) analysis of TCGA and CGGA datasets showing that genes which facilitate HDAC function are upregulated in IDH1/2^mut^ glioma. The top 15 GO terms are ranked based on Combined Score, and the GO term relating to HDAC activity is highlighted in yellow

First, gene expression profiles from The Cancer Genome Atlas (TCGA) and the Chinese Glioma Genome Atlas (CGGA) were analyzed [19, 20]. IDH1/2^wt^ and IDH1/2^mut^ glioma samples were matched by grade (Grade 2/3 versus Grade 4) and subsequently subjected to differential gene expression analysis. Differentially expressed genes (DEGs) were further evaluated via gene ontology (GO) analysis. In both Grade 4 datasets from TCGA and CGGA, IDH1/2 status was significantly associated with upregulation of genes that modulate HDAC activity (Fig. 1b, d). In the low grade glioma cohort, this effect was only observed in the TCGA dataset, though this was not statistically significant based on adjusted p-value estimates (Fig. 1a, c). Nevertheless, 8/11 of the HDAC-related genes identified via GO analysis are significantly upregulated in the low grade TCGA and CGGA datasets (Fig. S1). HDAC-related genes upregulated in IDH1/2^mut^ glioma include HDAC2/4/5, KDM1A, CHD4, MTA2/3, SIRT1/2, PHF21A, and BRMS1L. These data support the concept that IDH1/2^mut^ gliomas have increased activity in HDAC pathways, and suggests targeting histone acetylation via HDAC inhibitors may exhibit profound effects in IDH1/2^mut^ glioma.

### Characterization of IDH expression and activity in glioma cell lines

To assess the effect of HDAC inhibition on glioma based on IDH1/2 status, we employed six patient-derived glioma cell lines: three IDH1/2^wt^ (0827, 0923, 0211) and three IDH1^mut^ (0905, BT142, TB096). No patient-derived IDH2^mut^ glioma cell lines were used as they are extremely rare (only 2.3% of all glioma patients bear an IDH2^mut^) [21]. Of the three IDH1^mut^ glioma cell lines, two contain the canonical heterozygous R132H mutation (0905, TB096) while BT142 has a hemizygous R132H mutation, with the wild-type allele having been lost. Information on clinical pathology and other relevant genetic alterations, are provided in Table S1. IDH1 R132H expression was confirmed using western blot and reflected the genomic status for each line, with BT142 exhibiting enhanced production of the mutant R132H IDH1 enzyme (Fig. S2a). D2HG quantitation via ELISA also showed that 0905 and TB096 cell lines produced higher levels of D2HG, whereas BT142 more closely resembled IDH1/2^wt^ glioma cells (Fig. S2b), consistent with prior studies indicating that both IDH1^wt^ and IDH1^mut^ alleles are required for prominent D2HG production [8, 22–24].

### IDH1^mut^ glioma cells display increased cytotoxicity with HDAC inhibition

**Fig. 2:**
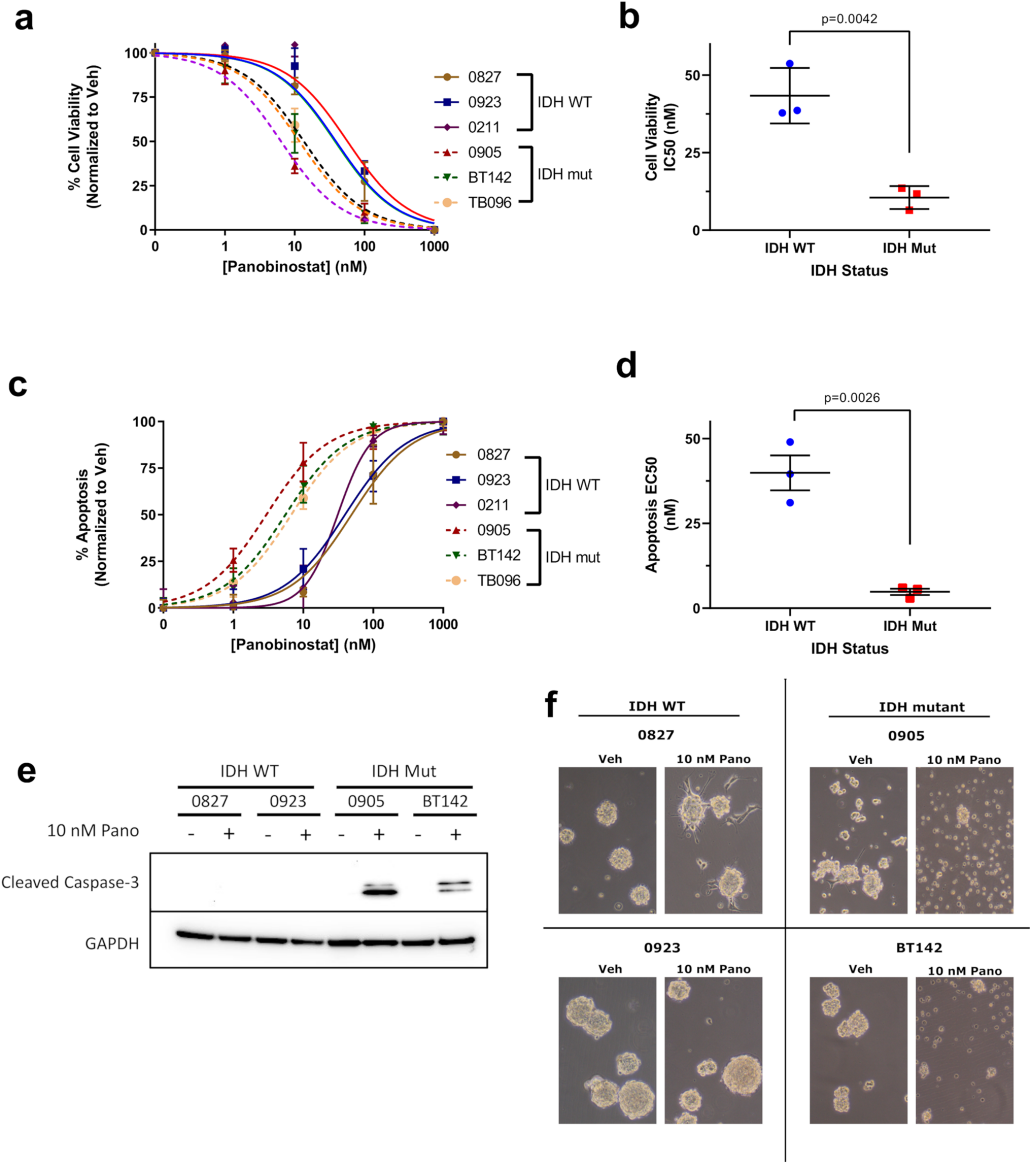
IDH1^mut^ glioma is particularly sensitive to the cytotoxic effects of panobinostat. **a** Normalized cell viability of IDH1/2^wt^ and mutant glioma cells treated with the HDAC inhibitor panobinostat for 5 days (n = 3). Nonlinear regression statistical analysis was utilized to determine IC50 values. **b** Consolidation of cell viability IC50 values derived from **a** stratified based on IDH status (n = 9). Statistical analysis was performed via Student’s t-test. **c** Induction of apoptosis in IDH1/2^wt^ and IDH1^mut^ glioma cells treated with panobinostat for 5 days (n = 3). Nonlinear regression statistical analysis was utilized to determine IC50 values. **d** Consolidation of apoptosis EC50 values derived from **c** stratified based on IDH status (n = 9). Statistical analysis was performed via Student’s t-test. **e** Western blot of cleaved caspase 3 in whole cell lysates extracted from IDH1/2^wt^ and IDH1^mut^ glioma cells treated with 10 nM panobinostat for 5 days. **f** Phase-contrast microscopy of IDH1/2^wt^ and IDH1^mut^ glioma cells treated with 10 nM panobinostat for 5 days

Viability of IDH1/2^wt^ and IDH1^mut^ glioma cells was determined using DNA chromophore cell viability and Annexin V apoptosis assays. In cell viability dose–response assays, IDH1^mut^ glioma cells were much more sensitive to an FDA-approved pan-HDACi, panobinostat, compared to their IDH1/2^wt^ counterparts, with a 4.1 fold difference in IC_50_ values (Fig. 2a, b; Fig. S3; Table S2). This response was not dependent on culture conditions, as IDH1^mut^ cells cultured in serum-free glioma stem cells (0905 and BT142) and in serum-containing media (TB096) showed the same heightened sensitivity. Annexin V apoptosis dose–response assays showed an even more pronounced sensitivity to panobinostat in IDH1^mut^ cells, with a tenfold difference in average EC_50_ values compared to IDH1/2^wt^ (Fig. 2c, d; Fig. S4; Table S2). (The disparity of magnitude between the apoptosis and cell viability data is likely due to the apoptosis assay detecting cells that were still viable, but were in early phases of apoptosis.) Western blot analysis showed selective induction of cleaved caspase-3 (CC3) exclusively in IDH1^mut^ glioma cells cultured with 10 nM panobinostat (Fig. 2e), confirming that panobinostat triggers apoptosis in these cells.

Furthermore, phase-contrast microscopy images showed that 0827 and 0923 IDH1/2^wt^ cells retained sphere size and number in culture when treated with 10 nM panobinostat for 5 days, whereas IDH1^mut^ cells exhibited visible signs of cytotoxicity, including reduction in sphere size, sphere number, and a pronounced increase in single cells that were phase-dark (Fig. 2f). To examine whether these data simply reflect a general chemosensitivity in IDH1^mut^ cells, both IDH1/2^wt^ and IDH1^mut^ cells were subjected to puromycin dose–response assays, which showed no difference in sensitivity based on IDH status (Fig. S5; Table S3). Together, these data suggest that IDH1^mut^ glioma cells are preferentially sensitive to the cytotoxic effects of the HDACi panobinostat.

### Panobinostat preferentially inhibits cell proliferation in IDH1^mut^ glioma cells

**Fig. 3:**
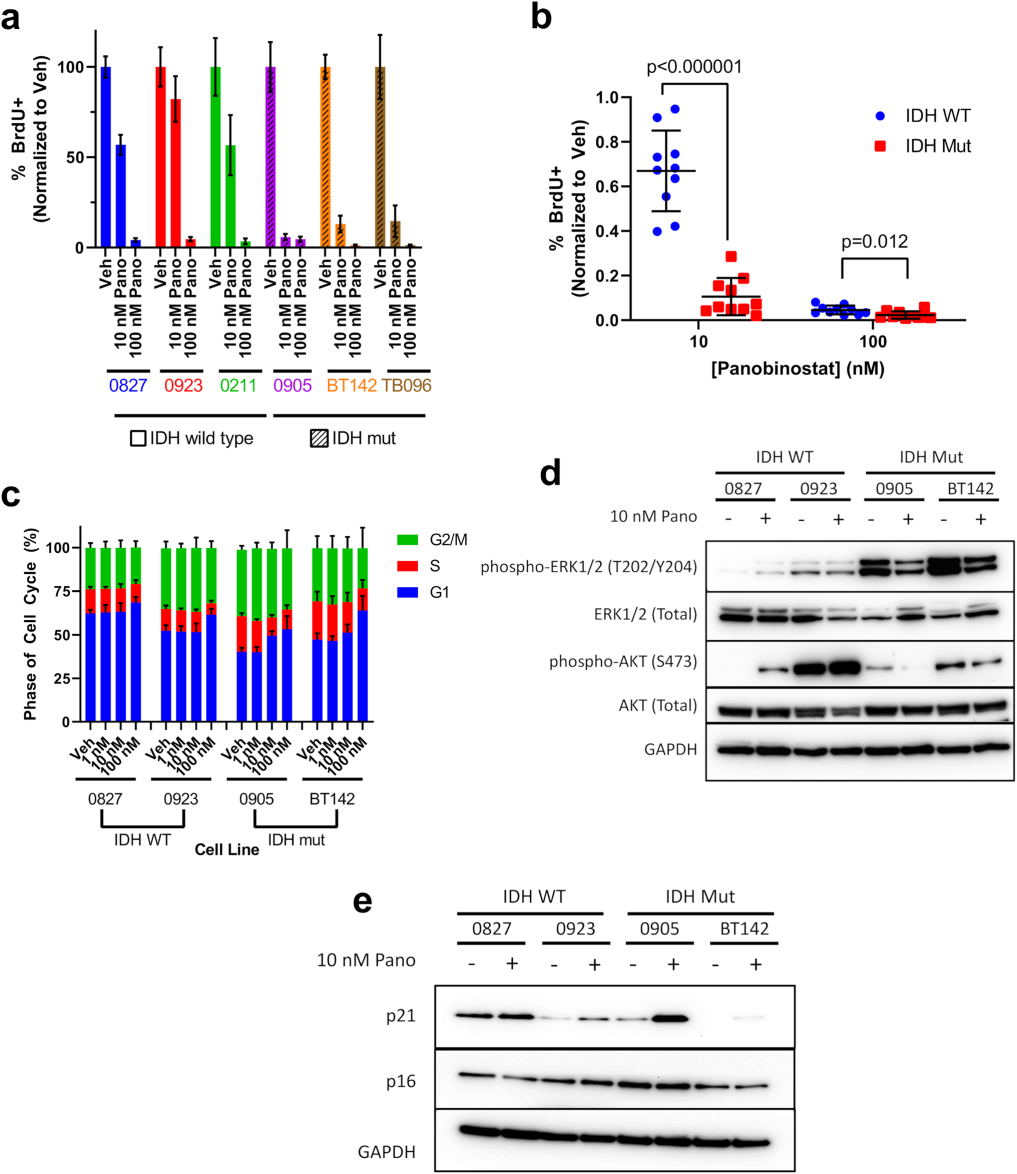
Panobinostat preferentially reduces proliferation in IDH1^mut^ glioma. **a** BrdU incorporation assays of IDH1/2^wt^ and IDH1^mut^ glioma treated with 10 nM and 100 nM panobinostat for 48 h (n = 3). **b** Consolidation of data from **a** stratified based on IDH status (n = 6 for IDH1/2^wt^ and n = 9 for IDH1^mut^). Statistical analysis was performed via Student’s t-test. **c** Cell cycle analysis of IDH1/2^wt^ and IDH1^mut^ glioma treated with 20 nM and 100 nM panobinostat for 48 h (n = 3). **d** Western blot of ERK and AKT in whole cell lysates extracted from IDH1/2^wt^ and IDH1^mut^ glioma cells treated with 10 nM panobinostat for 2 days. **e** Western blot of p21 and p16 in whole cell lysates extracted from IDH1/2^wt^ and IDH1^mut^ glioma cells treated with 10 nM panobinostat for 2 days

To determine the effect of panobinostat on IDH1/2^wt^ versus IDH1^mut^ glioma cell proliferation, cells were treated with panobinostat, then subjected to BrdU proliferation assays. Whereas 10 nM panobinostat suppressed IDH1/2^wt^ cell proliferation by 33% on average, it suppressed IDH1^mut^ proliferation by 89.5% (Fig. 3a, b; Fig. S6a, b). The difference was much smaller, though still statistically significant, at 100 nM panobinostat. Cell cycle analysis further supported the notion that IDH1^mut^ cells are more sensitive to panobinostat, with a statistically significant induction of G1 arrest/reduction in G2-M phase only observed in IDH1^mut^ cells (Fig. 3c; Fig. S7). Finally, 10 nM panobinostat suppressed the pro-proliferation MAPK and AKT signaling pathways in IDH1^mut^ cells, not IDH1/2^wt^ cells (Fig. 3d). Further, the cell cycle suppressor, p21, was variably induced by panobinostat in both IDH1^mut^ cell lines tested, and in only one of the IDH1/2^wt^ cell lines (Fig. 3e). No effects were seen on the related cell cycle inhibitor p16 (Fig. 3e). Together, these data suggest that, in addition to increased cytotoxicity, IDH1^mut^ glioma cells are also preferentially sensitive to the antiproliferative effects of HDACi.

### HDACi elicits a greater increase in histone H3 acetylation in IDH1^mut^ glioma cells

**Fig. 4:**
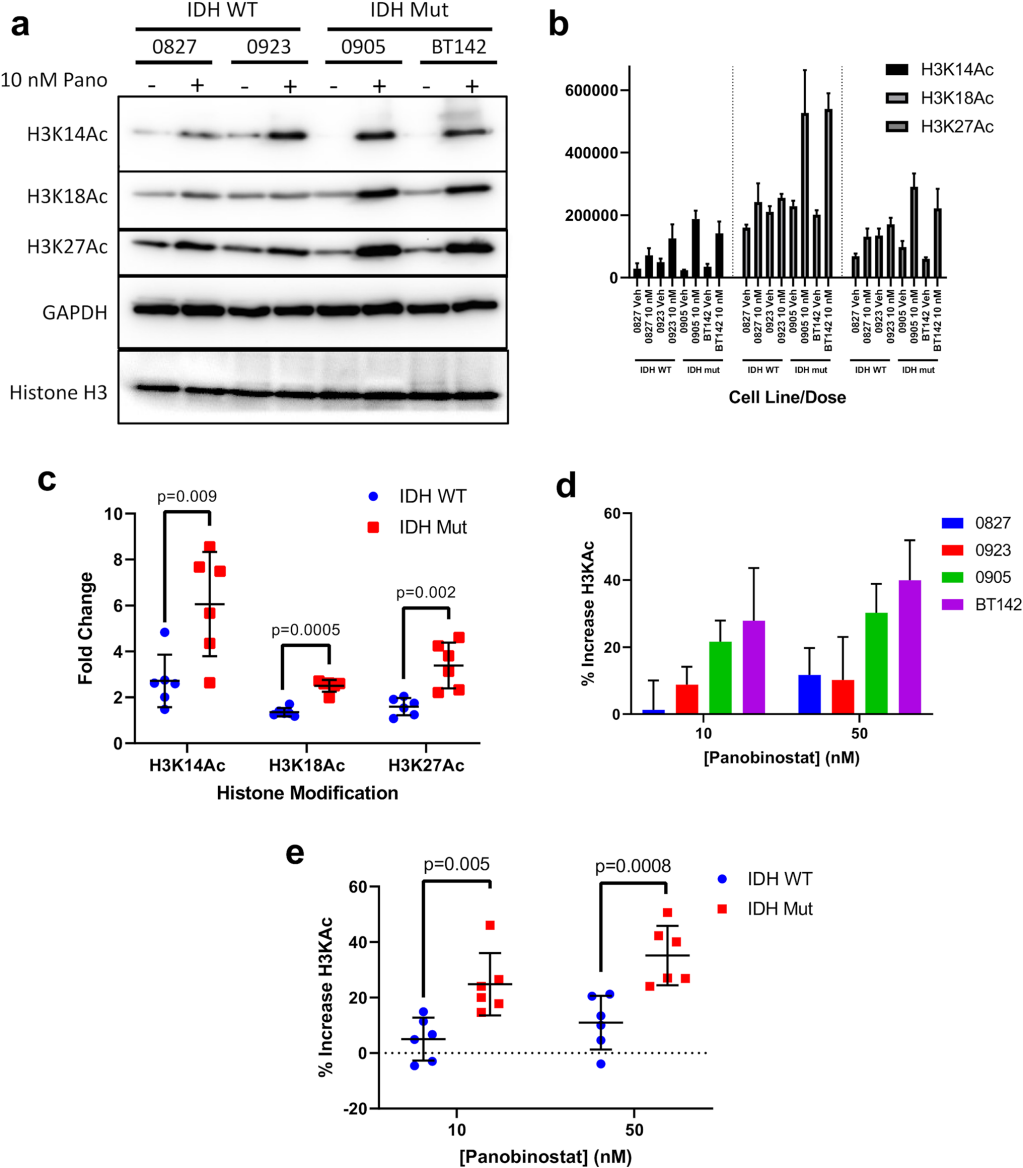
IDH1^mut^ glioma preferentially upregulates Histone H3 acetylation in response to panobinostat treatment. **a** Western blot of H3K14Ac, H3K18Ac, and H3K27Ac in whole cell lysates extracted from IDH1/2^wt^ and IDH1^mut^ glioma cells treated with 10 nM panobinostat for 2 days. **b** Densitometry analysis of H3K14Ac, H3K18Ac, and H3K27Ac western blots normalized to GAPDH. Whole cell lysates were extracted from IDH1/2^wt^ and IDH1^mut^ glioma cells treated with 10 nM panobinostat for 2 days. Plots show total signal based on three independent experiments. **c** Consolidation of data from **b** stratified based on IDH status (n = 6) showing fold change in H3K14Ac, H3K18Ac, and H3K27Ac in response to 10 nM panobinostat for 2 days. Statistical analysis was performed via Student’s t-test. **d** Total H3KAc levels analyzed via ELISA in nuclear extracts from IDH1/2^wt^ and IDH1^mut^ glioma cells treated with 10 and 50 nM panobinostat for 2 days (n = 3). **e** Consolidation of data from **d** stratified based on IDH status (n = 6). Statistical analysis was performed via Student’s t-test

Both IDH1/2^wt^ and IDH1^mut^ glioma cells responded to panobinostat with increased H3K14/18/27Ac histone marks (Fig. 4a). However, IDH1^mut^ cells showed a three to sixfold induction in those marks, compared to a twofold increase in IDH1/2^wt^ cells (Fig. 4b, c; Fig. S8). To evaluate global changes in histone acetylation, an ELISA against total H3KAc was used, which showed that total H3KAc content increased by 20% at 10 nM panobinostat, and 35% at 50 nM panobinostat, with IDH1^mut^ cells showing an approximate twofold increase in H3KAc compared to IDH1/2^wt^ cells (Fig. 4d, e). These data indicate that the heightened cytotoxic and antiproliferative effects by panobinostat in IDH1^mut^ glioma cells are associated with a preferential increase in acetylated chromatin.

### IDH1^mut^ glioma cells are also more sensitive to the HDACi valproic acid

**Fig. 5:**
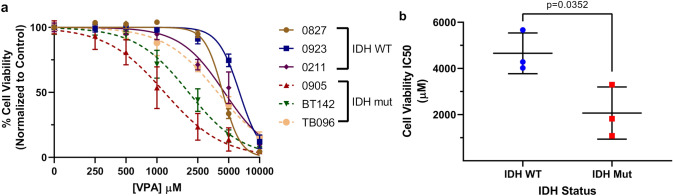
Valproic acid recapitulates the preferential cytotoxicity in IDH1^mut^ glioma. **a** Normalized cell viability of IDH1/2^wt^ and IDH1^mut^ glioma cells treated with the HDAC inhibitor valproic acid for 5 days (n = 3). Nonlinear regression statistical analysis was utilized to determine IC50 values. **b** Consolidation of cell viability IC50 values from **a** stratified based on IDH status (n = 9). Statistical analysis was performed via Student’s t-test

To investigate whether other HDACi are also preferentially active against IDH1^mut^ glioma cells, IDH1/2^wt^ and IDH1^mut^ cells were treated with valproic acid (VPA), an antiepileptic with HDACi properties [25]. As with panobinostat, VPA was more toxic to IDH1^mut^ cells than IDH1/2^wt^ cells, with an IC_50_ of ~ 2 mM for IDH1^mut^ cells versus 4.6 mM for IDH1/2^wt^ cells (Fig. 5, Fig. S9).

## Discussion

Despite the comparatively longer survival of patients with IDH1^mut^ gliomas versus IDH1/2^wt^ glioblastomas, IDH1^mut^ gliomas remain an ultimately fatal, incurable disease, and thus new treatment options are needed for these tumors. Targeting the canonical IDH1 R132H variant, both by small molecule inhibitors and by a vaccine, are in clinical trials [26–28]. Other therapeutic strategies exploiting potential metabolic vulnerabilities in IDH1^mut^ gliomas, including inhibitors of poly (ADP-ribose) polymerase and nicotinamide phosphoribosyltransferase, are also being pursued [29–31]. Yet, to date, comparatively little has been done to target the unique effects of IDH1^mut^ on the glioma epigenome and chromatin.

In this study, we showed that cultured IDH1^mut^ glioma cells are more sensitive than IDH1/2^wt^ cells to the FDA-approved pan-HDACi, panobinostat, and that this is mediated through both cytotoxic and cytostatic effects (Figs. 2 and 3). This contrasts with some other IDH1^mut^-specific cancer therapies, such as selective IDH1^mut^ inhibitors that suppress proliferation but are not cytotoxic [20]. Panobinostat was effective against IDH1^mut^ cells cultured in either serum free media (0905 and BT142) or serum-supplemented media (TB096), suggesting that the effect is not unique to a particular glioma cell type or culture condition. Even BT142 cells, which deleted the wild-type IDH1 allele and therefore no longer produce as much D2HG, were still more sensitive to panobinostat and therefore suggest a durable therapeutic response. This indicates that HDACi may still be a useful strategy against IDH1^mut^ gliomas that have decreased D2HG production during disease progression. Additionally, the ribosomal inhibitor, puromycin, was not more active against IDH1^mut^ glioma cells, demonstrating that the effects of panobinostat are unlikely to be the result of a more generalized chemosensitivity of IDH1^mut^ cells.

One key finding in this data is the greater upregulation of total histone H3 acetylation in IDH1^mut^ glioma cells treated with panobinostat, specifically in H3K14/18/27Ac active chromatin marks (Fig. 4). Such upregulation may be driving the increased sensitivity of IDH1^mut^ cells to HDACi. Our data suggests that this sensitivity may be due to enhanced regulation of the acetylome in IDH1^mut^ glioma due to increased HDAC activity (Fig. 1). Considering that panobinostat is a pan-HDACi, future experiments will investigate the role of specific HDAC isoforms in mediating increases in histone acetylation in response to panobinostat, as well as to determine the potential of HDACi against in vivo models of IDH1^mut^ gliomas.

In addition to their proven role in treating multiple myeloma [18], HDACi are being explored in a number of other solid tumors, including glioblastomas and diffuse intrinsic pontine gliomas [32–35]. However, results thus far in IDH1/2^wt^ glioblastoma patients have been disappointing [36–38]. One study that did include 10 IDH1^mut^ astrocytoma patients was inconclusive, perhaps in part because of a reliance on historical controls, and an exclusive focus on recurrent cases that had already been treated with standard therapy [39]. A recent study by others suggested that IDH1^mut^ gliomas were actually more resistant to HDACi [40]; however, that study used overexpression of IDH1 R132H in U87MG and U373MG glioblastoma cells. Our data demonstrate the importance of using patient-derived models, with endogenous IDH1^mut^, when studying therapeutics aimed at altering the epigenome and/or chromatin.

In this study, another HDACi, VPA, was also more effective against IDH1^mut^ glioma cells (Fig. 5). VPA has already been tested in clinical trials against IDH1/2^wt^ pediatric and adult gliomas, with varying results [41–43]. To date, however, it has not been tested specifically in patients with IDH1^mut^ gliomas. Considering that VPA is a powerful antiepileptic often used to control seizures in glioma patients, and that IDH1^mut^ gliomas are highly epileptogenic [44], VPA might have a dual purpose in IDH1^mut^ glioma patients.

In summary, we identified that cultured glioma cells with endogenous IDH1^mut^ are preferentially sensitive to the cytotoxic and antiproliferative effects of the FDA-approved HDACi, including panobinostat and valproic acid. HDACi increases the amount of acetylated chromatin in IDH1^mut^, providing a potential molecular rationale for this increased sensitivity to HDACi. In sum, this study suggests the potential of HDACi inhibition as a therapeutic strategy specifically for the treatment of IDH1^mut^ glioma.

## Materials and methods

### Cell lines

Glioma stem cells 0827, 0923, 0211 and 0905 were previously isolated at the Neuro-Oncology Branch, NIH and have been previously characterized for glioma stem-like properties and genomic alterations [22–25]. BT142 [26] was purchased from ATCC, while TB096 [11] was a kind gift from Dr. Hai Yan at Duke University. All cell lines were grown in medium that includes Neurobasal-A base medium, B-27 and N2 supplements along with EGF and FGF growth factors (NBE). Two cell lines, 0905 and BT142, required 100 ng/mL PDGF-AA in the medium. TB096 was cultured in half NBE and half DMEM with 10% FBS. All cell cultures were cultured at 37C and 5% CO2 with 1 × penstrep and were frequently monitored for mycoplasma contamination. Additionally, cells were cultured in suspension as neurospheres except for TB096 which exhibited adherent growth. Cell culture medium was changed 3x/week. Panobinostat (Fischer Scientific; NC0021665) was dissolved in 100% DMSO, sterile filtered, and diluted to 1000 × in DMSO for drug treatments. For all treatments, vehicle concentrations were 0.1% or less.

### Analysis of TCGA and CGGA Data

Gene expression data from TCGA and CGGA datasets was downloaded as raw counts and processed using the online tool Appyter [19, 20, 45]. DEGs between IDH^wt/mut^ gliomas were generated using the Limma package with a p-value cutoff of 0.05 and a fold change cutoff of 0.2. GO analysis employed the use of the online tool Enrichr using the top 1000 DEGs [45]. Gene expression plots were created by uploading data to the UCSC Xena Browser, organizing data by IDH status and tumor grade, and then generating grade- and IDH-matched gene expression boxplots with corresponding t-test statistics [46].

### Cell viability and apoptosis assays

Cell viability and apoptosis assays were conducted using a Luminex Guava® Muse® Cell Analyzer in conjunction with a Muse® Cell Count and Viability Kit or a Muse® Annexin V and Dead Cell Kit. Single-cell suspensions were analyzed on the Muse® Cell Analyzer according to the manufacturer’s protocols. Briefly, cells were trypsinized (0.05%) and then mixed with Cell Count and Viability reagent or 1:1 NBE to Annexin V and Dead Cell reagent, incubated for the specified amount of time on manufacturer’s protocol, and then inserted into the Muse® Cell Analyzer for data acquisition.

### Phase-contrast microscopy

Phase-contrast microscopy was performed using an Olympus CK40 inverted microscope with a Canon EOS 700D camera mounted via an OM-Mount Photomicro Adapter L. Briefly, cells were placed on the microscope stage, allowed to settle, and 2–3 images were taken of different fields of view. Images were captured using the EOS Utility software.

### Immunoblotting

Cell samples were washed with 1X DPBS, pelleted via centrifugation, flash-frozen in liquid nitrogen, and finally stored at − 80 °C until cell lysis. Total cell extracts were prepared by resuspending cell pellets in 1 × RIPA buffer containing 1 × protease and phosphatase inhibitor and then subjecting pellets to sonication for 3 × 20 s. Insoluble fractions were pelleted and discarded, and then subsequent protein quantitation was performed on clarified cell lysates via Bradford assay. SDS-PAGE and transfer steps were conducted via the Novex immunoblot system. Blots were visualized using a ProteinSimple Fluorchem® E Imager. Bands on immunoblots were quantified via AlphaView SA v3.5. Quantified immunoblots all experienced the same transfer conditions, primary/secondary antibody concentrations, and exposure times. Antibody information can be found in Supplementary Methods.

### Enzyme-linked immunosorbent assay (ELISA)

Detection of intracellular 2-HG levels was performed using the Abcam D-2-Hydroxyglutarate Assay kit (ab211070) according to the manufacturer’s protocol. Briefly, cells were trypsinized (0.05%), pelleted via centrifugation, and flash frozen in liquid nitrogen. Once ready for further analysis, cells were lysed according to manufacturer’s protocol, stably spotted on strip-wells, and then processed for final colorimetric analysis at 450 nm via a Molecular Devices SpectraMax® 340 microplate reader.

Detection of total histone H3 acetylation was performed via Epigentek’s EpiQuik® Global Histone H3 Acetylation Assay kit according to the manufacturer’s protocol. Briefly, histones were acid extracted, stably spotted on strip-wells, and antibodies bound to antigen. Finally, samples were analyzed colorimetrically at 450 nm and quantified via standard curve.

### Cell cycle analysis

Cells were analyzed for changes in cell cycle distribution via Muse® Cell Cycle kit according to manufacturer’s protocol. Briefly, cells were trypsinized (0.05%) to form a single cell suspension, washed with 1 × DPBS, and fixed in 70% ethanol at − 20 °C overnight. Fixed cells were then incubated with Muse® Cell Cycle reagent for 30 min at room temp away from light and then injected onto the Muse Cell Analyzer.

### BrdU incorporation assays

Cells were analyzed for BrdU incorporation via BD Pharmingen® BrdU Flow kit according to the manufacturer’s protocol. Briefly, cells were treated for 48 h with drug and then pulsed with 10 µM BrdU (except for our No BrdU control) for 2.5 h before washing, fixing, and storing at − 80 °C in solution of 1:9 FBS to DMSO. When ready for analysis, cells were then thawed, permeabilized, and incubated with anti-BrdU antibody according to manufacturer’s protocol. Once stained cell samples and unstained controls were ready for analysis, data was acquired using a Beckman Coulter Cytomics® FC500 flow cytometer with FITC filter applied. Cells were gated using the No BrdU control and then treated samples were assessed for BrdU-positive cells within this gate (Supplementary Figure).

### Statistical analyses

Student’s t test and nonlinear regression statistical analyses were performed via GraphPad Prism 8.0 software package. All replicate analyses are presented as mean ± standard deviation of three independent replicates at a significance level (α) of 0.05 unless otherwise indicated in the figure captions.

## Supplementary Information

Below is the link to the electronic supplementary material.

Supplementary file 1 Fig. S1 Gene expression of HDAC-related genes identified as significantly upregulated in IDH1/2mut glioma based on GO analysis. Statistical analyses were performed via Welch’s T-test. **a**, **b** Expression of GO-identified genes that promote HDAC function in Grade 2/3 IDH1/2mut glioma using samples from TCGA and CGGA datasets. **c**, **d** Expression of GO-identified genes that promote HDAC function in Grade 4 IDH1/2mut glioma using samples from TCGA and CGGA datasets (TIF 17402 kb)

Supplementary file 2 (TIF 18530 kb)

Supplementary file 3 Fig. S2 Glioma cell characterization. **a** Table showing known genetic alterations in glioma cells utilized in this study. Studies with analysis of genetic alterations are cited in the table. NIF = no information found. **b** Western blot of R132H IDH1 in whole cell lysates extracted from IDH1/2wt (0827, 0923, 0211) and IDH1mut (0905, BT142, TB096) glioma cells grown in NBE medium. **c** 2-HG levels assessed by ELISA in IDH1/2wt and IDH1mut glioma cells used in this study (TIF 24802 kb)

Supplementary file 4 Fig. S3 Cell viability dose-response assay with puromycin in IDH1/2wt and IDH1mut glioma cells. **a** Normalized cell viability of IDH1/2wt and IDH1mut glioma cells treated with puromycin for 3 days (n = 3). Nonlinear regression statistical analysis was utilized to determine IC50 values. **b** Consolidation of cell viability IC50 values derived from **a** stratified based on IDH status (n = 6). Statistical analysis was performed via Student’s t-test. **c** Representative Muse® cell analyzer flow cytometry plots from **a** using the Muse® Cell Count and Viability Kit. Y-axis represents uptake of a membrane permeable DNA dye by all cells, whereas the x-axis represents uptake of a membrane impermeable DNA dye e.g. 7-AAD in dead cells that have lost membrane integrity (TIF 44121 kb)

Supplementary file 5 Fig. S4 Representative Muse® cell analyzer flow cytometry plots from Fig. 2a, b using the Muse® Cell Count and Viability Kit. Y-axis represents uptake of a membrane permeable DNA dye by all cells, whereas the x-axis represents uptake of a membrane impermeable DNA dye e.g. 7-AAD in dead cells that have lost membrane integrity (TIF 44121 kb)

Supplementary file 6 Fig. S5 Representative Muse® cell analyzer flow cytometry plots from Fig. 2c, d using the Muse® Annexin V and Dead Cell Kit. Y-axis represents uptake of a membrane impermeable DNA dye e.g. 7-AAD in dead cells that have lost membrane integrity, whereas the x-axis represents binding of Annexin V to phosphatidyl-serine residues (TIF 19243 kb)

Supplementary file 7 Fig. S6 Flow cytometry plots from Fig. 3a, b BrdU incorporation assays. Plots were generated using the FCSalyzer v0.9.17 software program. BC = Background Control, Veh = Vehicle Control. **a** Representative bivariate forward- and side-scatter plots showing the gating method for our WT and mutant glioma cells. **b** Representative univariate plots (FITC) showing BrdU uptake in our IDH1/2wt and IDH1mut glioma cells. Included background stain controls did not receive BrdU treatment but were still stained with anti-BrdU antibody (TIF 44121 kb)

Supplementary file 8 (TIF 48340 kb)

Supplementary file 9 Fig. S7 Representative Muse® cell analyzer flow cytometry plots from Fig. 3c using the Muse® Cell Cycle Kit. DNA content on the x-axis is used as an indicator for cell cycle phase (TIF 27083 kb)

Supplementary file 10 Fig. S8 Validation of linearity with our antibodies used for quantitative immunoblot analysis. H3K27Ac shows issues with linearity, but the H3K27Ac signal from our quantitative experiments does not extend past the linear range. Linearity was determined using linear regression analysis via Graphpad Prism 8.0.0 (TIF 18111 kb)

Supplementary file 11 Fig. S9 Representative Muse® cell analyzer flow cytometry plots from Fig. 5a, b using the Muse® Cell Count and Viability Kit. Y-axis represents uptake of a membrane permeable DNA dye by all cells, whereas the x-axis represents uptake of a membrane impermeable DNA dye e.g. 7-AAD in dead cells that have lost membrane integrity (TIF 59441 kb)

Supplementary file 12 Table S1 Table showing known genetic and molecular alterations in the glioma cells used in this study with references to sources in the literature (PNG 105 kb)

Supplementary file 13 Table S2 Table showing IC50 and EC50 values from our cell viability and apoptosis studies in Fig. 2 (PNG 76 kb)
