# Supplementary figures and images for: IDH1 mutant glioma is preferentially sensitive to the HDAC inhibitor panobinostat
Source: J Neurooncol. 2021 Aug 23;154(2):159–70. doi: 10.1007/s11060-021-03829-0 (PMC8437887; doi:10.1007/s11060-021-03829-0)

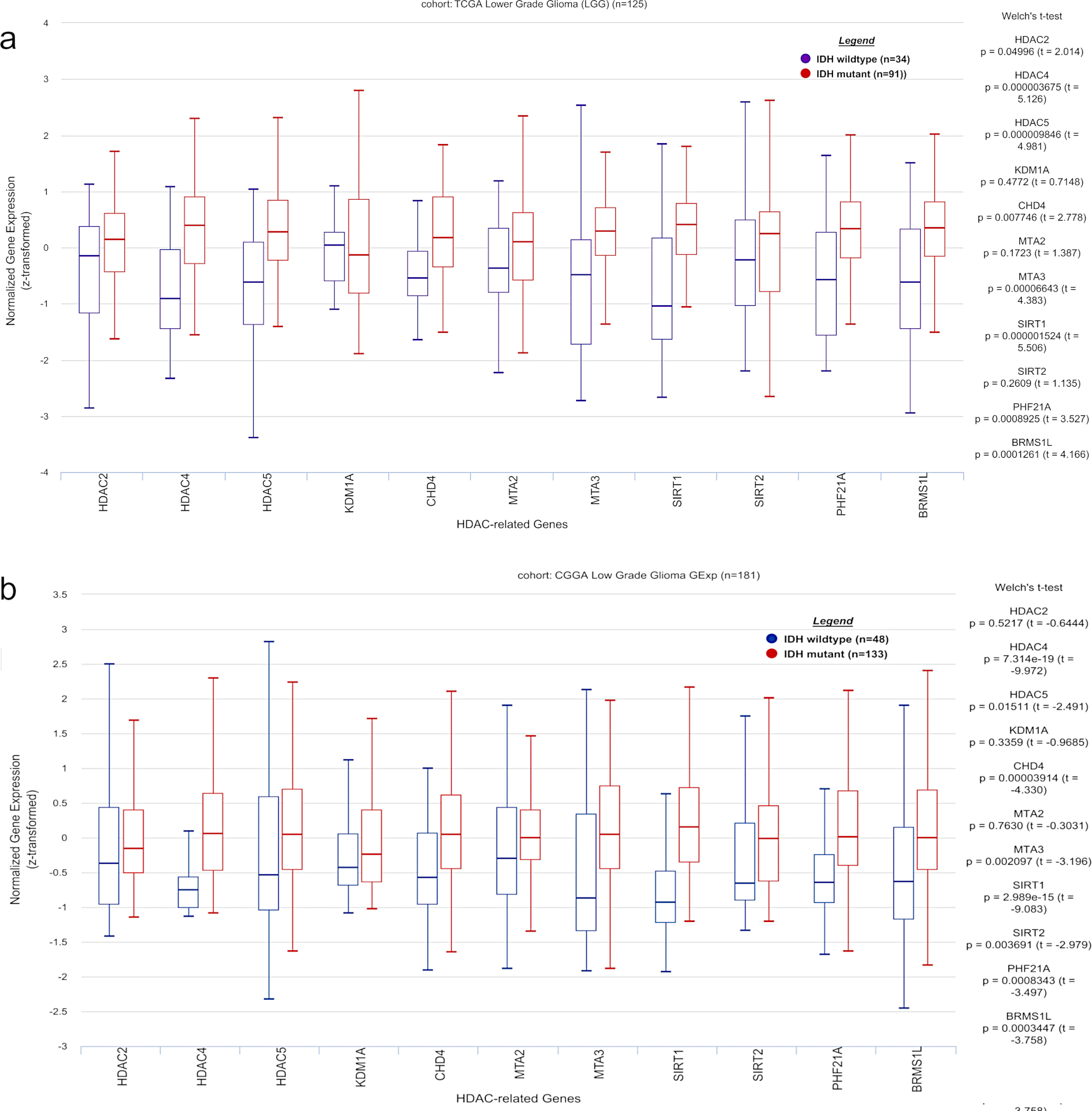

Supplement: Supplementary file 1 — Fig. S1 Gene expression of HDAC-related genes identified as significantly upregulated in IDH1/2mut glioma based on GO analysis. Statistical analyses were performed via Welch’s T-test. a, b Expression of GO-identified genes that promote HDAC function in Grade 2/3 IDH1/2mut glioma using samples from TCGA and CGGA datasets. c, d Expression of GO-identified genes that promote HDAC function in Grade 4 IDH1/2mut glioma using samples from TCGA and CGGA datasets (TIF 17402 kb) [file 11060_2021_3829_MOESM1_ESM.tif]

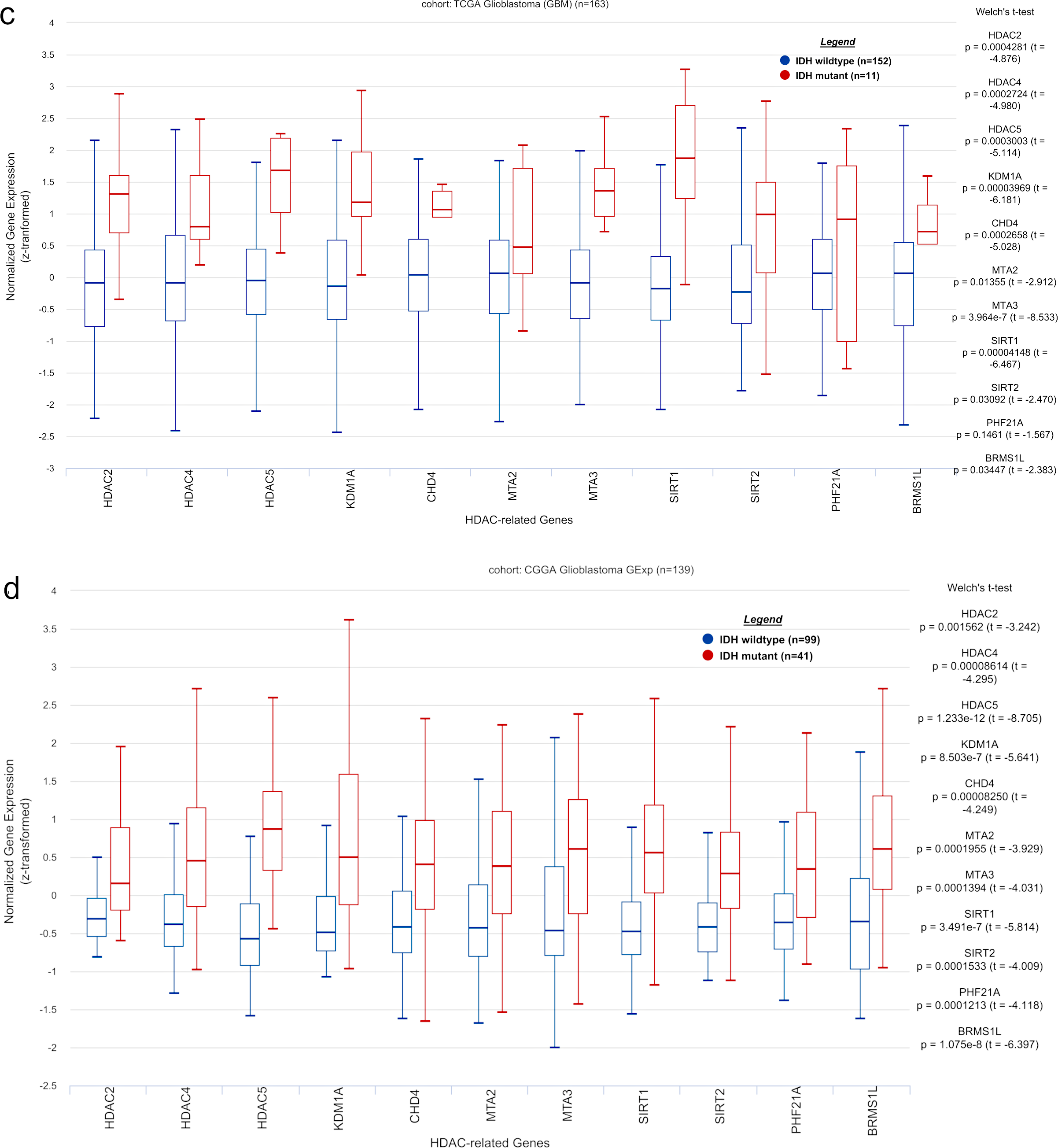

Supplement: Supplementary file 2 — (TIF 18530 kb) [file 11060_2021_3829_MOESM2_ESM.tif]

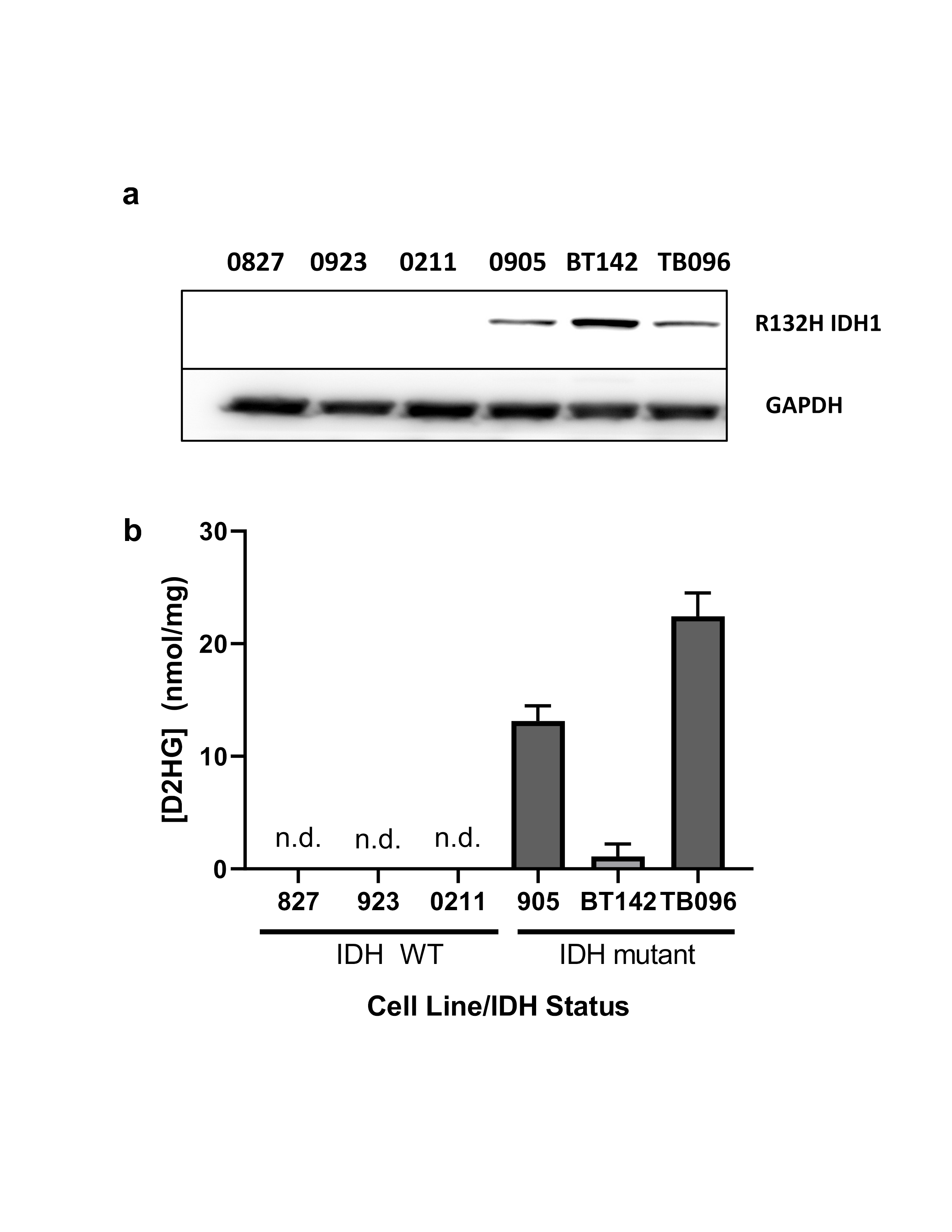

Supplement: Supplementary file 3 — Fig. S2 Glioma cell characterization. a Table showing known genetic alterations in glioma cells utilized in this study. Studies with analysis of genetic alterations are cited in the table. NIF = no information found. b Western blot of R132H IDH1 in whole cell lysates extracted from IDH1/2wt (0827, 0923, 0211) and IDH1mut (0905, BT142, TB096) glioma cells grown in NBE medium. c 2-HG levels assessed by ELISA in IDH1/2wt and IDH1mut glioma cells used in this study (TIF 24802 kb) [file 11060_2021_3829_MOESM3_ESM.tif]

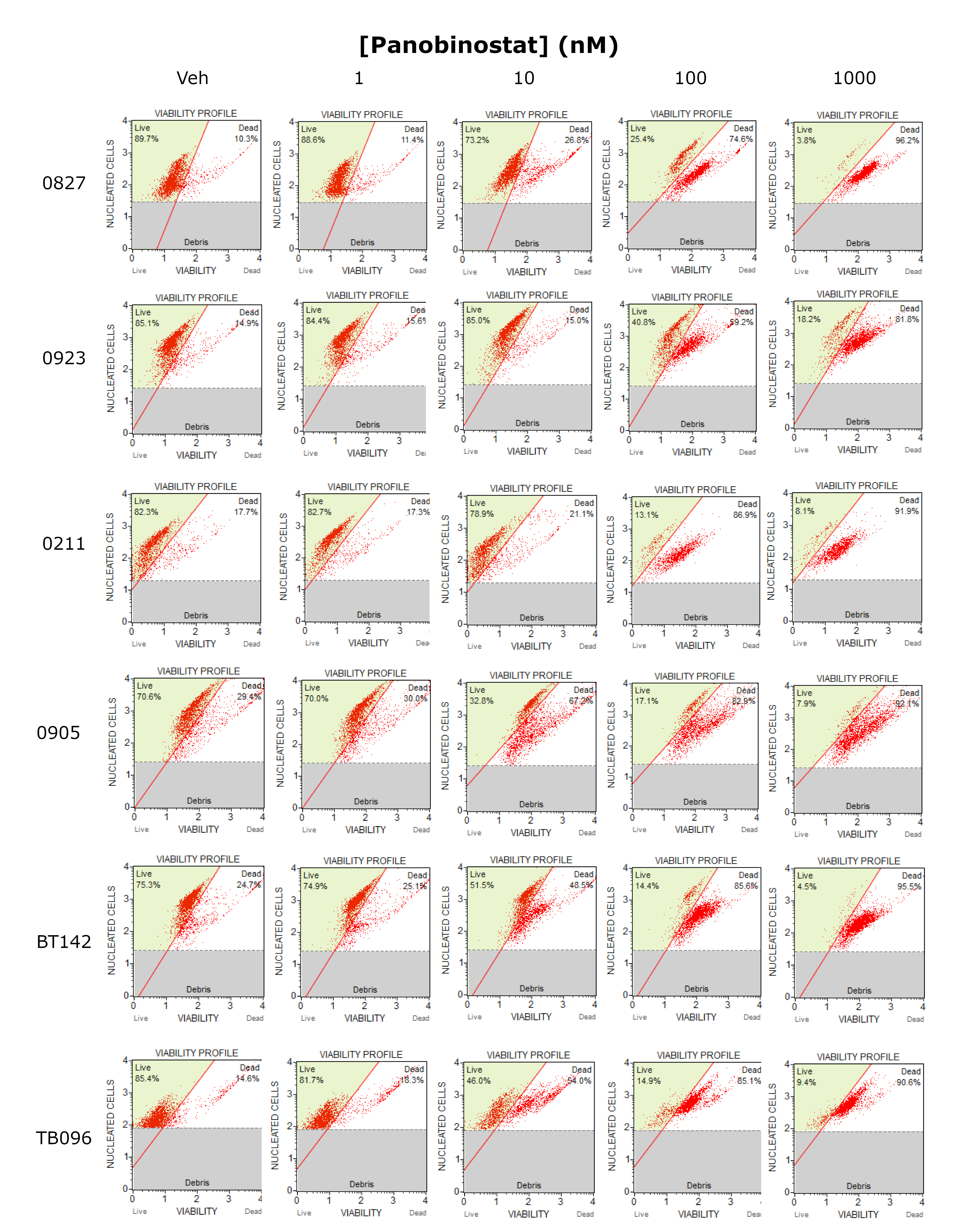

Supplement: Supplementary file 4 — Fig. S3 Cell viability dose-response assay with puromycin in IDH1/2wt and IDH1mut glioma cells. a Normalized cell viability of IDH1/2wt and IDH1mut glioma cells treated with puromycin for 3 days (n = 3). Nonlinear regression statistical analysis was utilized to determine IC50 values. b Consolidation of cell viability IC50 values derived from a stratified based on IDH status (n = 6). Statistical analysis was performed via Student’s t-test. c Representative Muse® cell analyzer flow cytometry plots from a using the Muse® Cell Count and Viability Kit. Y-axis represents uptake of a membrane permeable DNA dye by all cells, whereas the x-axis represents uptake of a membrane impermeable DNA dye e.g. 7-AAD in dead cells that have lost membrane integrity (TIF 44121 kb) [file 11060_2021_3829_MOESM4_ESM.tif]

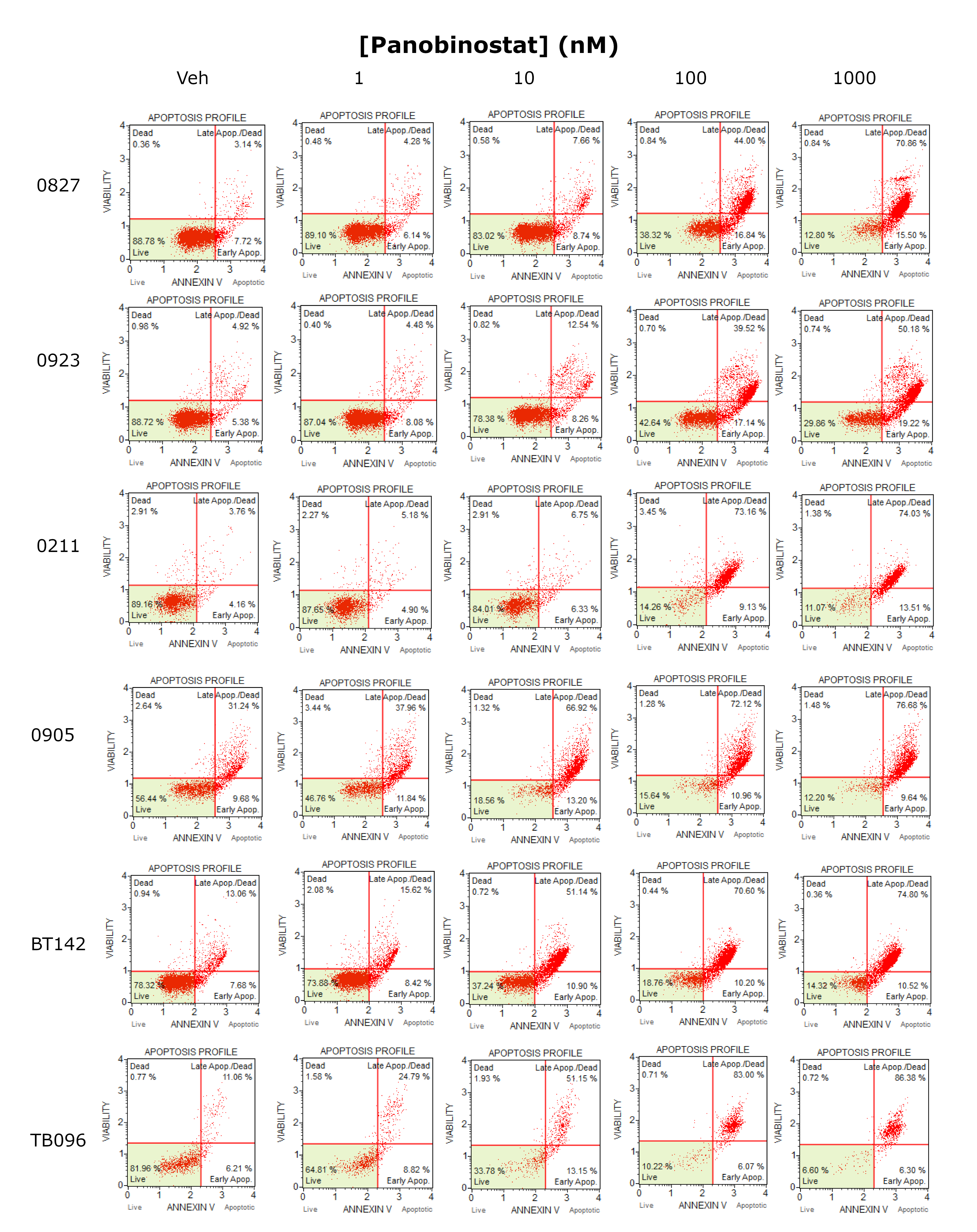

Supplement: Supplementary file 5 — Fig. S4 Representative Muse® cell analyzer flow cytometry plots from Fig. 2a, b using the Muse® Cell Count and Viability Kit. Y-axis represents uptake of a membrane permeable DNA dye by all cells, whereas the x-axis represents uptake of a membrane impermeable DNA dye e.g. 7-AAD in dead cells that have lost membrane integrity (TIF 44121 kb) [file 11060_2021_3829_MOESM5_ESM.tif]

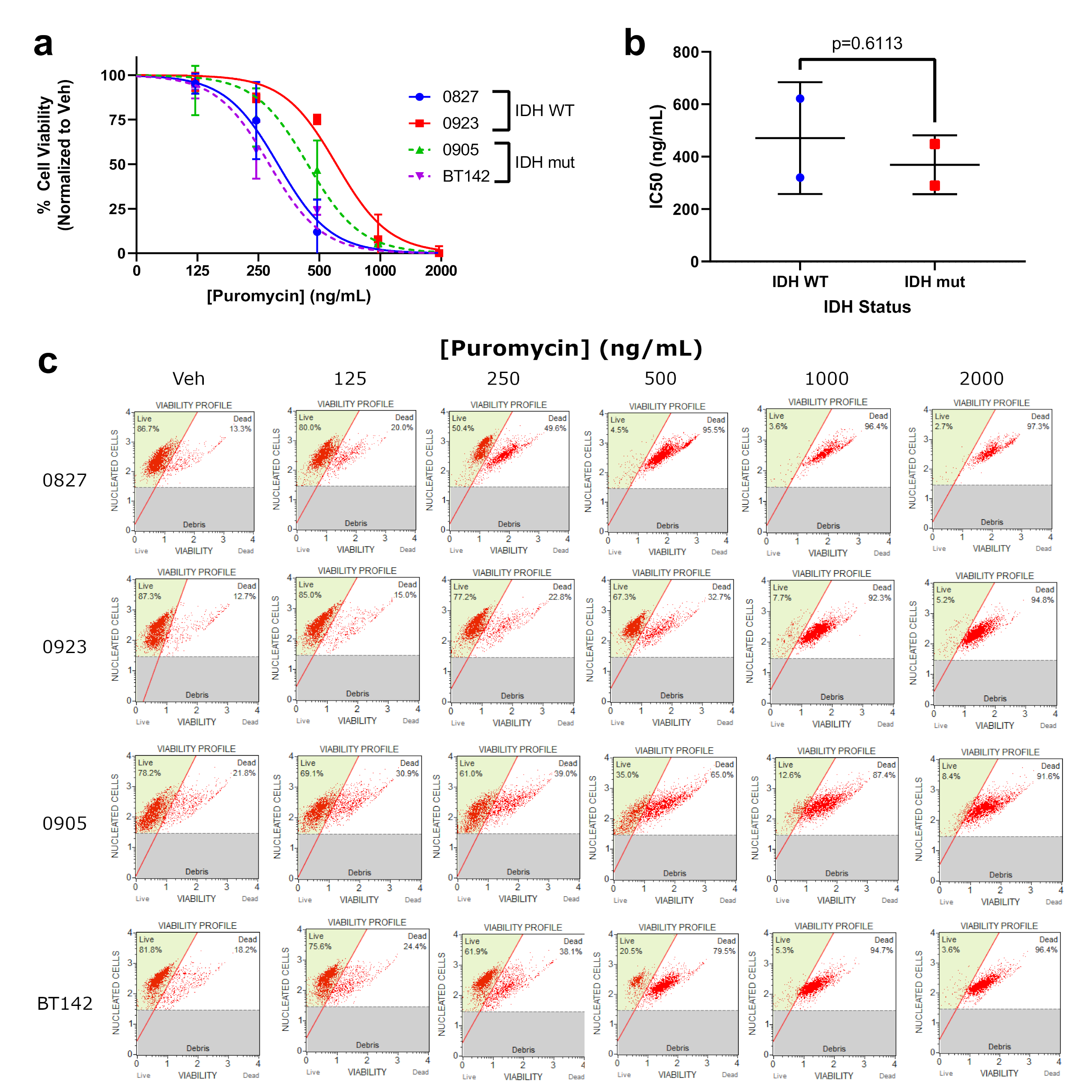

Supplement: Supplementary file 6 — Fig. S5 Representative Muse® cell analyzer flow cytometry plots from Fig. 2c, d using the Muse® Annexin V and Dead Cell Kit. Y-axis represents uptake of a membrane impermeable DNA dye e.g. 7-AAD in dead cells that have lost membrane integrity, whereas the x-axis represents binding of Annexin V to phosphatidyl-serine residues (TIF 19243 kb) [file 11060_2021_3829_MOESM6_ESM.tif]

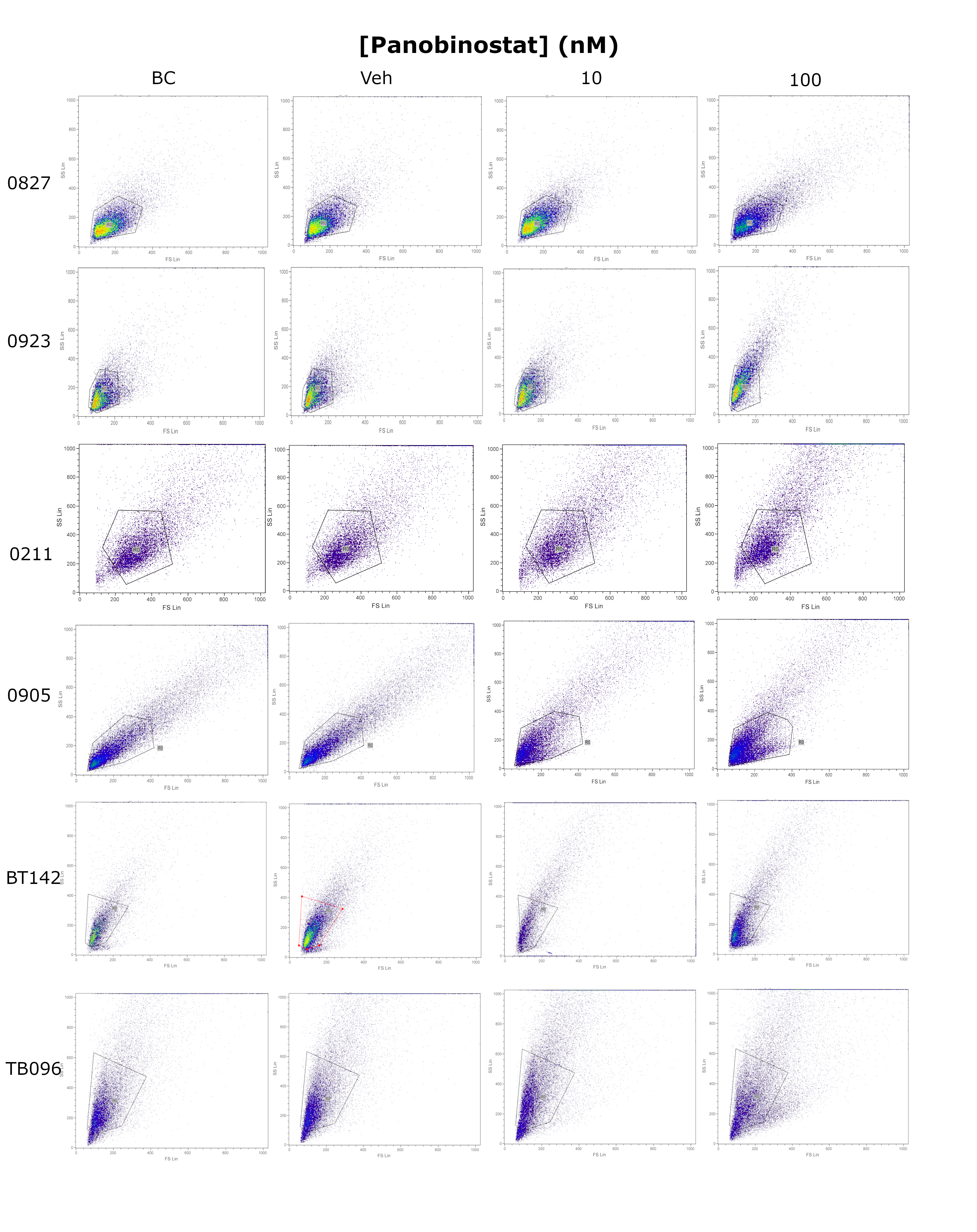

Supplement: Supplementary file 7 — Fig. S6 Flow cytometry plots from Fig. 3a, b BrdU incorporation assays. Plots were generated using the FCSalyzer v0.9.17 software program. BC = Background Control, Veh = Vehicle Control. a Representative bivariate forward- and side-scatter plots showing the gating method for our WT and mutant glioma cells. b Representative univariate plots (FITC) showing BrdU uptake in our IDH1/2wt and IDH1mut glioma cells. Included background stain controls did not receive BrdU treatment but were still stained with anti-BrdU antibody (TIF 44121 kb) [file 11060_2021_3829_MOESM7_ESM.tif]

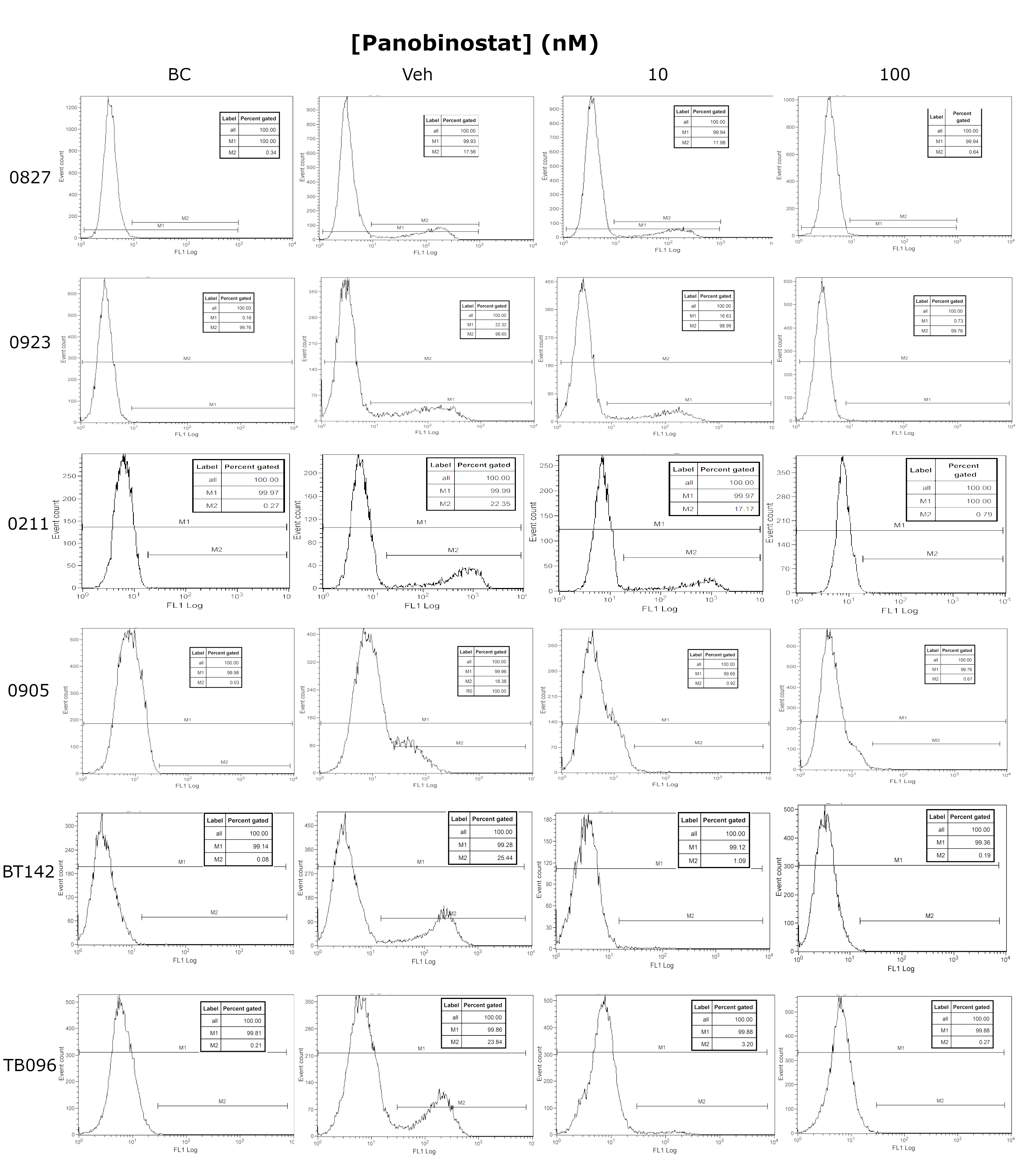

Supplement: Supplementary file 8 — (TIF 48340 kb) [file 11060_2021_3829_MOESM8_ESM.tif]

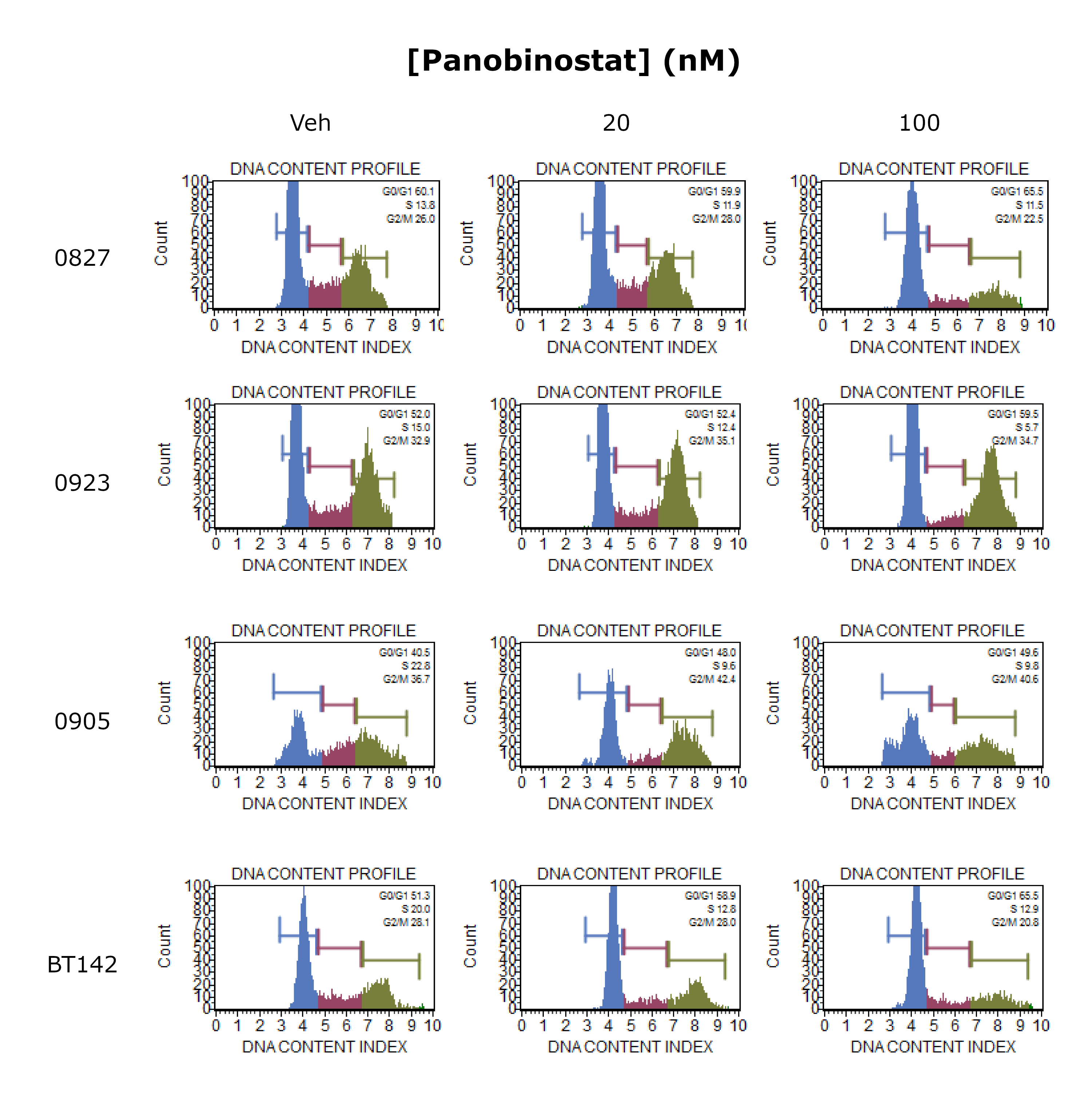

Supplement: Supplementary file 9 — Fig. S7 Representative Muse® cell analyzer flow cytometry plots from Fig. 3c using the Muse® Cell Cycle Kit. DNA content on the x-axis is used as an indicator for cell cycle phase (TIF 27083 kb) [file 11060_2021_3829_MOESM9_ESM.tif]

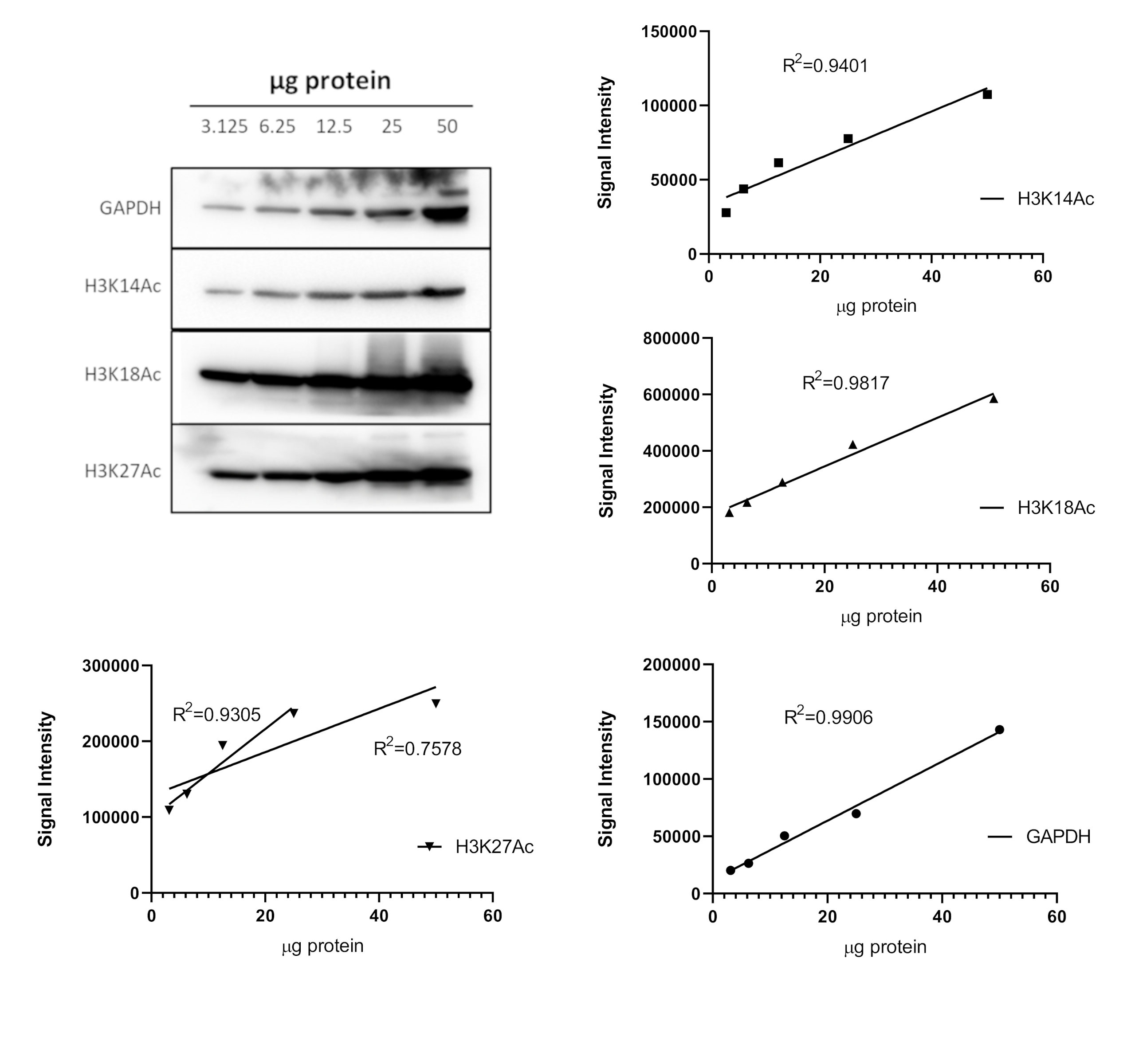

Supplement: Supplementary file 10 — Fig. S8 Validation of linearity with our antibodies used for quantitative immunoblot analysis. H3K27Ac shows issues with linearity, but the H3K27Ac signal from our quantitative experiments does not extend past the linear range. Linearity was determined using linear regression analysis via Graphpad Prism 8.0.0 (TIF 18111 kb) [file 11060_2021_3829_MOESM10_ESM.tif]

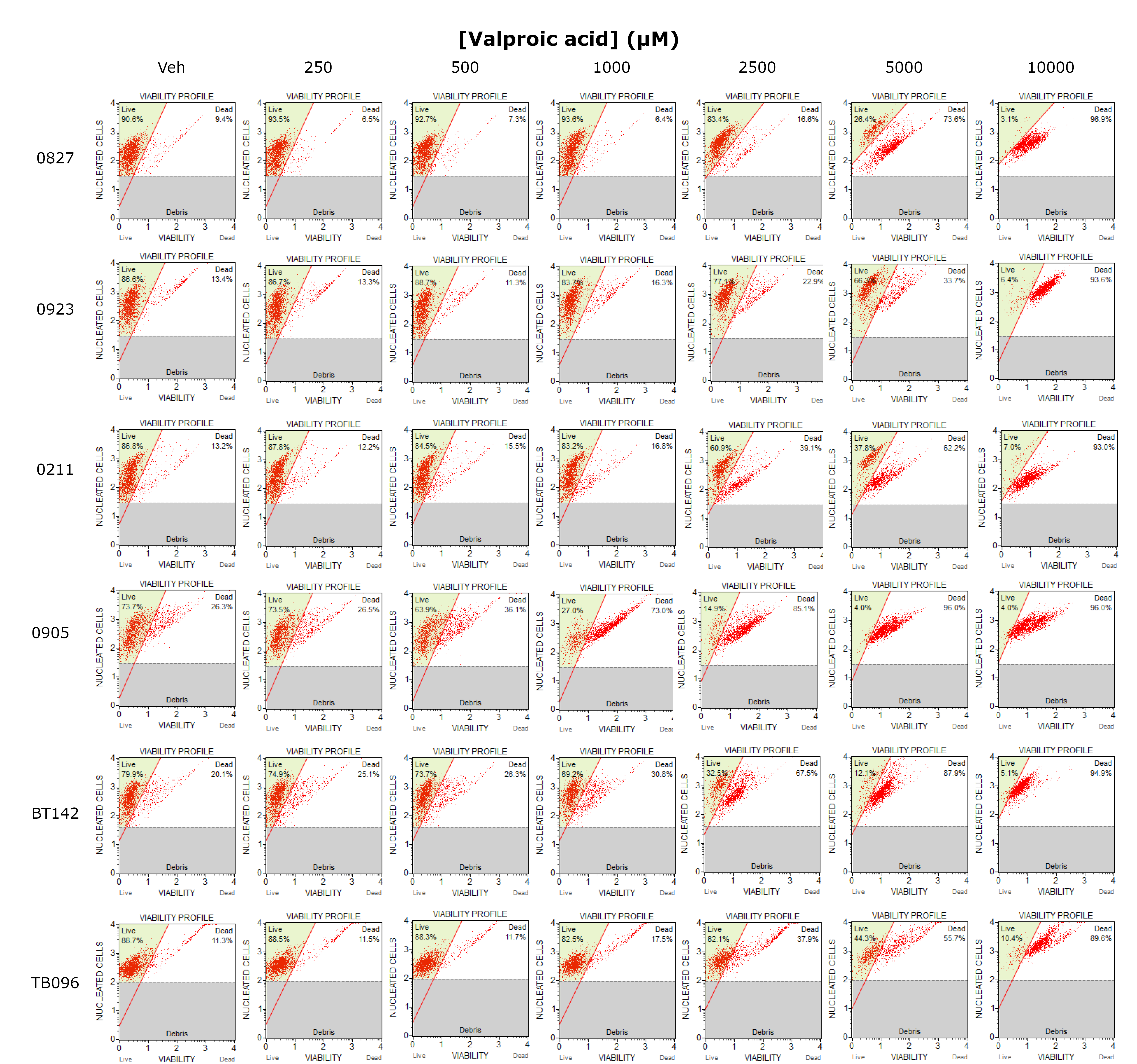

Supplement: Supplementary file 11 — Fig. S9 Representative Muse® cell analyzer flow cytometry plots from Fig. 5a, b using the Muse® Cell Count and Viability Kit. Y-axis represents uptake of a membrane permeable DNA dye by all cells, whereas the x-axis represents uptake of a membrane impermeable DNA dye e.g. 7-AAD in dead cells that have lost membrane integrity (TIF 59441 kb) [file 11060_2021_3829_MOESM11_ESM.tif]

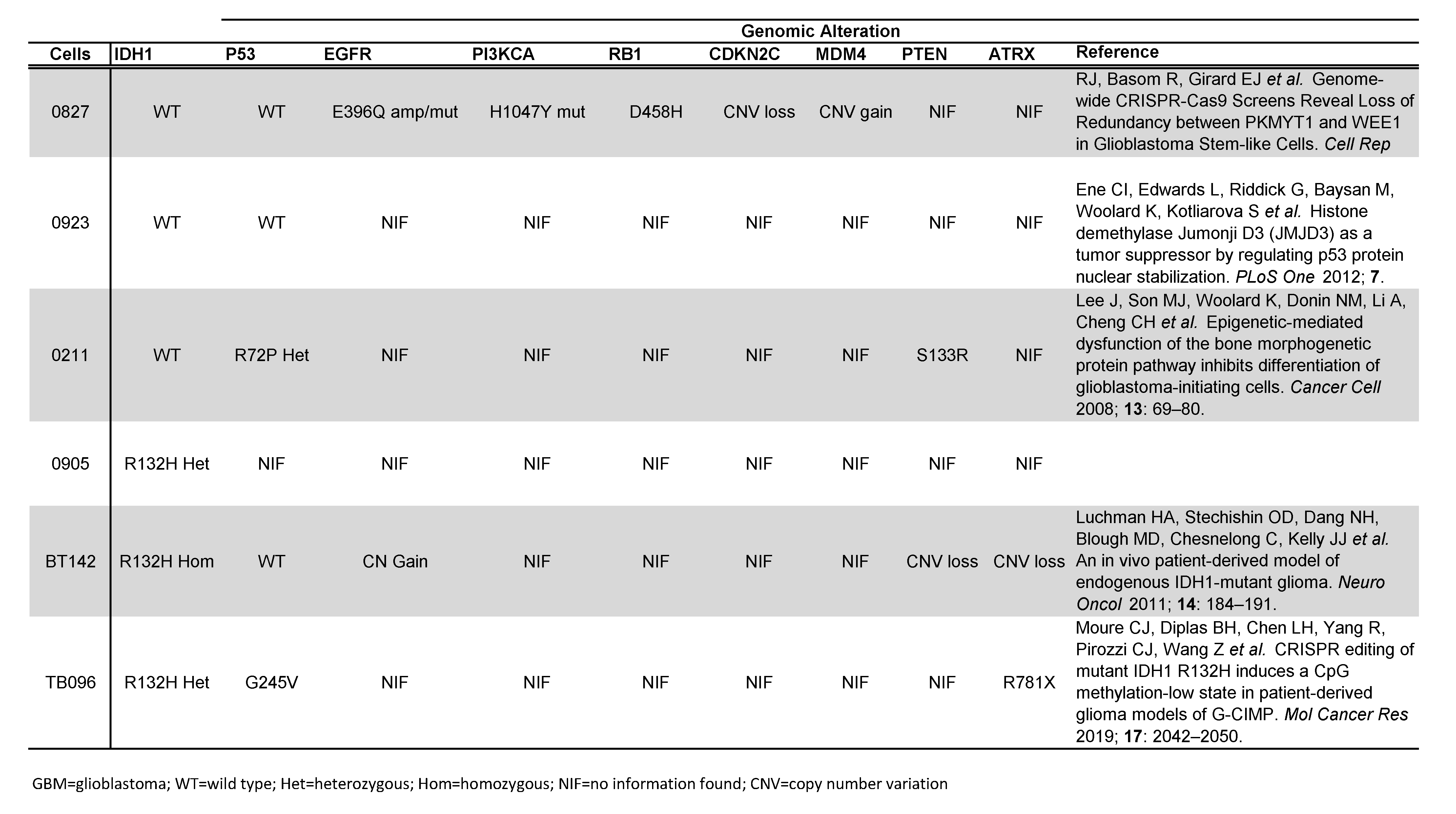

Supplement: Supplementary file 12 — Table S1 Table showing known genetic and molecular alterations in the glioma cells used in this study with references to sources in the literature (PNG 105 kb) [file 11060_2021_3829_MOESM12_ESM.png]

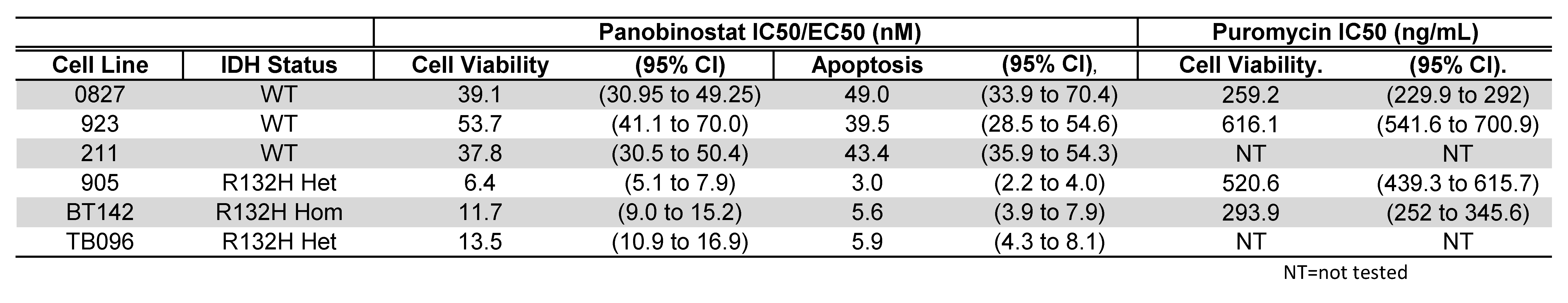

Supplement: Supplementary file 13 — Table S2 Table showing IC50 and EC50 values from our cell viability and apoptosis studies in Fig. 2 (PNG 76 kb) [file 11060_2021_3829_MOESM13_ESM.png]
